# Induction of labor in women with prior cesarean section: outcomes in a selected low-risk population

**DOI:** 10.1007/s00404-026-08436-w

**Published:** 2026-04-29

**Authors:** Sven Kehl, Christel Weiss, Hanna Düster, Simon Bader, Florian Faschingbauer, Michael Schneider, Matthias W. Beckmann, Ulf Dammer, Jutta Pretscher

**Affiliations:** 1 Department of Obstetrics and Gynecology, LMU University Hospital, LMU Munich, Campus Großhadern und Innenstadt, Marchioninistr. 15, 81377 Munich, Germany; 2 Department of Obstetrics and Gynecology, Erlangen University Hospital, Erlangen, Germany; 3 Department of Medical Statistics and Biomathematics, University Medical Center Mannheim, Heidelberg University, Mannheim, Germany; 4 Department of Obstetrics and Gynaecology, St. Theresien Hospital, Nuremberg, Germany

**Keywords:** TOLAC, VBAC, Labor induction, Cesarean section, Uterine rupture, Composite outcome, Maternal morbidity, Neonatal outcome

## Abstract

**Purpose:**

To investigate the impact of a previous cesarean section on maternal and perinatal outcomes in term pregnancies undergoing labor induction.

**Methods:**

In this retrospective cohort study, women with singleton, low-risk term pregnancies and labor induction were compared according to the presence or absence of a previous cesarean delivery. The primary outcome was a composite of adverse maternal and perinatal events. Secondary outcomes included cesarean section rate, mode of vaginal delivery, and specific maternal or neonatal complications.

**Results:**

The rate of composite adverse outcomes was comparable between groups (21.9% vs. 23.7%, *p* = 0.4826). However, placental abruption (1.3% vs. 0.3%, *p* = 0.0251), suspected triple I (1.9% vs. 0.4%, *p* = 0.0040), and shoulder dystocia (2.3% vs. 0.8%, *p* = 0.0265) occurred more frequently in women with a previous cesarean section. Abnormal cardiotocography (27.1% vs. 20.4%, *p* = 0.0058), operative vaginal delivery (17.8% vs. 11.9%, *p* = 0.0052), umbilical artery pH < 7.10 (4.9% vs. 2.8%, *p* = 0.0381), and the need for fetal blood sampling (8.7% vs. 5.0%, *p* = 0.0055) were also more common in this group. There was no difference in neonatal unit transfer (10.0% vs. 11.5%, *p* = 0.4328) or low Apgar scores (< 5 at 5 min: 0.3% vs. 0.4%, *p* = 1.0000). Cesarean section rates were similar (14.8% vs. 14.9%, *p* = 0.9692). In multivariable analysis, absence of prior vaginal delivery (OR = 3.460, *p* < 0.0001), higher maternal BMI (OR = 1.038, *p* < 0.0001), and older maternal age (OR = 1.033, *p* = 0.0002) were independently associated with adverse outcomes, whereas previous cesarean section was not.

**Conclusion:**

Labor induction in women with a prior cesarean section was not associated with increased risk for composite adverse maternal or perinatal outcomes. Nonetheless, TOLAC should be conducted in settings with immediate access to obstetric and neonatal intervention.

## What does this study add to the clinical work

**Table:** 

Induction of labor after previous cesarean section in a strictly selected low-risk population is not associated with an increased risk of composite adverse maternal or perinatal outcomes. However, these pregnancies require continuous surveillance and should only be managed in settings with immediate access to obstetric intervention.	

## Introduction

The global rise in cesarean section rates over recent decades has become a significant concern in obstetric care. Current estimates place the worldwide cesarean section rate at approximately 21%, with national rates as high as one-third in some countries, including Germany [1–3]. In nearly 30% of repeat cesarean section cases, the sole indication is a previous cesarean delivery [4]. Given the well-documented risks associated with multiple cesarean deliveries—such as uterine rupture, hemorrhage, and surgical complications—reducing primary and repeat cesarean section rates is a recognized international health priority.

Promoting trial of labor after cesarean (TOLAC) represents one strategy to mitigate these risks [5–9]. However, up to 27% of women attempting TOLAC require induction of labor (IOL) [10–14]. In these cases, individualized counseling is critical, particularly regarding the risk of uterine rupture, which is estimated to be between 0.5 and 1% under IOL conditions [15–17]. Current guidelines emphasize that TOLAC should only be offered in facilities with immediate access to emergency obstetric care.

Although spontaneous labor in women with a previous cesarean section is associated with higher success rates, IOL in this population remains common. Success rates for vaginal birth after cesarean (VBAC) following induction range from 60 to 85%, though the overall cesarean delivery rate tends to be higher than with spontaneous onset [17–19].

Despite the clinical relevance, data on adverse maternal and perinatal outcomes following IOL in women with a prior cesarean section remain limited [20].

While previous studies have primarily compared TOLAC with spontaneous labor or elective repeat cesarean delivery, the specific impact of induction of labor in women with a uterine scar remains less well defined. In clinical practice, however, once the decision for induction has been made, the relevant question is whether the presence of a prior cesarean section alters the maternal and perinatal risk profile under otherwise comparable conditions. Therefore, this study aims to specifically assess the outcomes of induction of labor in women with and without a prior cesarean section.

## Materials and methods

The study population was identified from a prospectively maintained institutional database of all deliveries at a single tertiary referral center and included women with singleton pregnancies in cephalic presentation at ≥ 37 + 0 weeks of gestation who underwent induction of labor (IOL) between January 2013 and December 2022. Missing data were minimal and handled by complete-case analysis.

Women with gestational diabetes, intrahepatic cholestasis of pregnancy, hypertensive disorders, abnormal findings in cardiotocography or fetal Doppler examinations, oligohydramnios, intrauterine growth restriction, large-for-gestational-age fetuses, intrauterine fetal demise, structural or chromosomal fetal anomalies, or placenta previa were excluded. Additionally, women who underwent elective repeat cesarean delivery or had a history of more than one prior cesarean section were not included. The study cohort thus comprised women without further obstetric risk factors apart from a previous cesarean section.

Data were collected during routine clinical care and anonymized prior to analysis. Due to the retrospective study design, informed consent was not required, and approval was granted by the local clinical ethics committee (No. 23-480-Br). Gestational age was based on the last menstrual period and adjusted using first-trimester crown–rump length measurements in cases with a discrepancy greater than seven days. The Bishop score was routinely assessed by experienced midwives or obstetricians.

The standard protocol for IOL generally began with the insertion of a double-balloon catheter (CRB, Cook® Medical) in women with an unfavorable cervix, filled with 80 mL saline per balloon according to the manufacturer’s instructions. The catheter’s external end was fixed to the patient’s inner thigh. After 12 to 24 h, the catheter was removed, and the Bishop score was reassessed. If cervical ripening remained insufficient, pharmacologic agents were used based on the Bishop score. Misoprostol (oral or vaginal) and dinoprostone (gel or insert) were administered according to institutional protocols. Balloon catheters were not used in cases with prelabor rupture of membranes. In cases of a favorable cervix (Bishop score > 6), oxytocin was initiated under continuous cardiotocography monitoring. Amniotomy was not performed routinely.

Women with a history of cesarean section were counseled regarding the off-label status of all pharmacologic induction methods in this context, in line with national guidelines. Detailed information was provided about the risks and benefits of labor induction versus elective repeat cesarean delivery, and written informed consent was obtained. In this subgroup, cervical ripening was initiated with CRB. If further induction was necessary, vaginal dinoprostone gel was used (standard dose: 2 mg, repeated after six hours if Bishop score < 6; maximum daily dose: 4 mg). In women with a favorable cervix (Bishop score > 6), intravenous oxytocin was administered with continuous fetal monitoring. Misoprostol was not used in women with a prior cesarean section due to safety considerations. As a result, pharmacologic induction strategies differed between women with and without a prior cesarean section. Amniotomy was not routinely performed.

Indications for induction of labor followed institutional standards and primarily included common low-risk clinical scenarios such as prelabor rupture of membranes, post-term pregnancy, and maternal request. The choice of induction method was based on a standardized protocol, primarily guided by cervical status (Bishop score), with consistent application across both study groups. Thus, clinical management was comparable between groups, minimizing potential bias related to treatment heterogeneity.

Cardiotocography (CTG) was performed for 30 min before induction and after CRB insertion. In women with a history of cesarean section, continuous CTG monitoring was maintained from the onset of regular contractions until delivery. After the administration of misoprostol or dinoprostone gel, CTG was monitored for 60 min and repeated every two hours. For dinoprostone inserts, CTG was conducted every three hours for 30 min or upon onset of contractions. Oxytocin administration was always accompanied by continuous monitoring.

All outcome variables were predefined and consistently extracted from the clinical database according to institutional standards. The primary outcome was a composite of adverse maternal and perinatal outcomes, including arterial umbilical cord pH < 7.00, base excess < −16, 5-min Apgar score < 5, neonatal intensive care unit transfer, uterine rupture, and placental abruption. Secondary outcomes included cesarean delivery rate, mode of vaginal delivery (spontaneous vs. operative), meconium-stained amniotic fluid, abnormal CTG (FIGO score), fetal blood sampling, suspected triple I (signs of intrauterine infection, maternal fever in combination with additional clinical parameters), and umbilical cord pH < 7.10 with base excess < −12. Outcome variables were defined according to standard clinical criteria routinely applied in obstetric practice.

The composite outcome was defined to capture the overall maternal and perinatal risk associated with induction of labor. Given the low incidence of individual adverse events, the use of a composite endpoint was intended to increase statistical power and provide a more comprehensive assessment of clinically relevant complications.

The study was conducted and reported in accordance with STROBE recommendations.

### Statistical analyses

All statistical analyses were performed using SAS software, release 9.4 (SAS Institute Inc., Cary, North Carolina, USA). For qualitative factors, absolute frequencies and percentage values are given. Quantitative variables are presented as mean values with standard deviations. In order to compare two groups regarding a qualitative factor, Chi2 test was used. Only if the assumptions of this test were not fulfilled, Fisher’s exact test was used instead. For comparing of two means, a two-sample t-test was been performed. As the data of these variables (i.e. age, BMI) are symmetrically distributed and because of the rather high sample sizes we assumed that the requirements of the t-test were not violated. For the adverse composite outcome several univariable logistic regression analyses were performed and an Odds Ratio was assessed for each impact factor. Furthermore, a multiple logistic regression analysis was performed in order to analyze several variables simultaneously using the SAS “backward selection”. In general, the result of a statistical test was considered as statistically significant for *p* < 0.05.

## Results

During the study period, a total of 25,196 deliveries were recorded. After applying inclusion and exclusion criteria, 3,393 women who underwent IOL at term were eligible for analysis. Of these, 3,083 women had no history of cesarean section, while 310 women had a previous cesarean delivery.

### Baseline characteristics

Demographic parameters are presented in Table 1. Women in the previous cesarean section group were significantly older (33.4 ± 4.7 vs. 31.1 ± 5.0 years, *p* < 0.0001) and more frequently lacked a prior vaginal delivery (79.3% vs. 72.6%, *p* < 0.0001) compared to women without a previous cesarean section. Other parameters such as BMI, gestational age, fetal birth weight, and prelabor rupture of membranes (PROM) did not differ significantly between groups.

**Table 1:** Demographic parameters

	No previous cesarean section group (n = 3083)	Previous cesarean section group (n = 310)	*p*-Value
Maternal Age (years)	31.1 ± 5.0	33.4 ± 4.7	< 0.0001
No previous vaginal delivery (n, %)	2238 (72.6%)	245(79.3%)	< 0.0001
BMI (kg/m^2^)	29.0 ± 5.6	29.3 ± 5.4	0.3362
Gestational age (days)	282.1 ± 8.9	282.6 ± 8.6	0.3729
Fetal weight (gram)	3480.9 ± 409.7	3519.2 ± 437.9	0.1184
Prelabor rupture of the membranes (n, %)	1573 (51.0%)	165 (53.2%)	0.4593

Quantitative variables are presented as mean ± standard deviation; for qualitative factors absolute frequencies and percentages are given. In order to compare two mean values, 2 sample t test has been applied. For qualitative factors, Chi^2^ test has been used. If the preconditions of a Chi^2^ test have not been fulfilled, Fisher’s exact test has been used instead

### Maternal and perinatal outcomes

The rate of the composite outcome did not differ significantly between the two groups (21.9% vs. 23.7%, *p* = 0.4826) (Table 2). There were no cases of uterine rupture or maternal death in either group. However, placental abruption occurred more frequently in the previous cesarean section group (1.3% vs. 0.3%, *p* = 0.0251), as did suspected triple I (1.9% vs. 0.4%, *p* = 0.0040), abnormal CTG (27.1% vs. 20.4%, *p* = 0.0058), and fetal blood sampling (8.7% vs. 5.0%, *p* = 0.0055).

**Table 2:** Outcome measures

	No previous cesarean section group (n = 3.083)	Previous cesarean section group (n = 310)	*p*-Value
Adverse composite outcome (n, %)	731 (23.7%)	68 (21.9%)	0.4826
Neonatal death (n, %)	2 (0.06%)	0	1.0000
Maternal death (n, %)	0	0	n.c
Cesarean section (n, %)	460 (14.9%)	46 (14.8%)	0.9692
pH < 7.00	12 (0.4%)	0	0.6171
Base excess < − 16	9 (0.3%)	1 (0.3%)	1.0000
Apgar score after 5 min < 5 (n, %)	13 (0.4%)	1 (0.3%)	1.0000
Transfer to neonatal care unit (n, %)	354 (11.5%)	31 (10.0%)	0.4328
Uterine rupture (n, %)	0	0	n.c
Placental abruption (n, %)	9 (0.3%)	4 (1.3%)	0.0251
Mode of delivery			
Vaginal delivery (n, %)	2623 (85.1%)	264 (85.2%)	0.9692
Mode of vaginal delivery			
Spontaneous delivery	2312 (88.1%)	217 (82.2%)	0.0052
Vaginal operative delivery	311 (11.9%)	47 (17.8%)	
pH < 7.10	85 (2.8%)	15 (4.9%)	0.0381
Base excess < −12	75 (2.4%) (n = 3074)	7 (2.3%)	0.8428
Apgar score after 5 min	9.6 ± 0.9	9.4 ± 1.0	0.0095
Meconium stained amniotic liquor (n, %)	400 (13.0%)	45 (14.5%)	0.4434
Abnormal cardiotocography (n, %)	629 (20.4%)	84 (27.1%)	0.0058
Fetal blood analysis (n, %)	154 (5.0%)	27 (8.7%)	0.0055
Suspected triple I (n, %)	12 (0.4%)	6 (1.9%)	0.0040
Shoulder dystocia (n, %)	26 (0.8%)	7 (2.3%)	0.0265

n.c., not calculable

Arterial umbilical cord pH < 7.10 occurred more frequently in the previous cesarean section group (4.9% vs. 2.8%, *p* = 0.0381), and shoulder dystocia occurred more frequently (2.3% vs. 0.8%, *p* = 0.0265). The mean 5-min Apgar score was slightly lower in women with a prior cesarean sections (9.4 ± 1.0 vs. 9.6 ± 0.9, *p* = 0.0095). However, severe neonatal outcomes such as Apgar < 5 at 5 min, pH < 7.00, base excess < −12 or < −16, neonatal deaths, and NICU transfers did not differ significantly between groups.

### Mode of delivery and cesarean indications

The overall cesarean section rate was nearly identical between groups (14.8% vs. 14.9%, *p* = 0.9692). Among those who achieved vaginal delivery, operative vaginal birth was more common in the previous cesarean section group (17.8% vs. 11.9%, *p* = 0.0052). The main indications for cesarean section were abnormal CTG and arrest of labor, with no significant differences between groups (Table 3).

**Table 3:** Indication for cesarean section when labor was induced

	No previous cesarean section group (n = 238)	Previous cesarean section group (n = 40)	*p*-Value
Abnormal cardiotocography (n, %)	107 (45.0%)	19 (47.5%)	0.1666
Abnormal fetal blood analysis (n, %)	14 (5.9%)	2 (5.0%)	
Arrest of labor (n, %)	104 (43.7%)	13 (32.5%)	
Suspected placental abruption (n, %)	4 (1.7%)	1 (2.5%)	
Suspected uterine rupture (n, %)	0	0	
Suspected triple I (n, %)	8 (3.4%)	5 (12.5%)	
Others (n, %)	1 (0.4%)	0	

### Regression analysis

In both univariable and multiple logistic regression analyses of the composite adverse outcome (Table 4), a prior cesarean section was not associated with a significantly increased risk (OR 1.106, *p* = 0.4826). In contrast, the absence of a prior vaginal delivery emerged as a strong independent predictor (univariable OR 3.254, multiple OR 3.460; both *p* < 0.0001). Other independent predictors included higher maternal BMI (multiple OR 1.038, *p* < 0.0001) and maternal age (multiple OR 1.033, *p* = 0.0002). Maternal age acted as a suppressor variable, enhancing the predictive validity of the variable “no prior vaginal delivery.” PROM, gestational age, and fetal birth weight were not significantly associated with the composite outcome in either model.

**Table 4:** Univariable and multiple regression analysis of the primary outcome parameter “adverse composite Outcome”

	Univariable analysis, odds ratio	Univariable *p* values	Multiple analysis, odds ratio	Multiple analysis, *p* values (significant factors)
No previous cesarean section group versus previous cesarean group	1.106	0.4826		
No previous vaginal delivery versus previous vaginal delivery	3.254	< 0.0001	3.460	< 0.0001
Body mass index (kg/m^2^)	1.035	< 0.0001	1.038	< 0.0001
Maternal age (years)	1.012	0.1586	1.033	0.0002
PROM (yes versus no)	1.1014	0.2332		
Gestational age (weeks)	1.040	0.2206		
Birth weight (kg)	1.044	0.6603		

## Discussion

In this study, we evaluated the maternal and perinatal outcomes of induction of labor in women with and without a prior cesarean section. No significant differences were observed in the composite outcome between the two groups. In contrast to previous studies focusing on the decision between TOLAC and elective repeat cesarean delivery, our analysis specifically addresses the effect of induction of labor itself. This approach allows a more focused evaluation of how a uterine scar modifies the risk profile under standardized induction conditions and may therefore provide additional information for clinical decision-making once induction is indicated.

The use of a composite outcome represents both a strength and a limitation of this study. While it allows a more comprehensive assessment of overall risk and improves statistical power for relatively rare events, it combines outcomes of different clinical relevance. Therefore, the composite endpoint should be interpreted alongside the individual outcome components, which are reported separately to allow a more nuanced clinical interpretation.

No cases of uterine rupture occurred in either group. This aligns with findings from Locatelli et al., who showed no increased risk of uterine rupture with IOL in women with prior cesarean section [10]. Stock et al. reported a uterine rupture rate of 0.66% in a large cohort of 7401 women with a previous cesarean section undergoing IOL at ≥ 39 weeks [21], while Ouzounian et al. found similar rupture rates between IOL and spontaneous labor (1.2% vs. 1.0%, *p* = 0.55) [22]. In contrast, Rossi et al. reported a higher rupture risk in IOL versus spontaneous labor in women with previous cesarean section (1.1% vs. 0.6%; OR: 1.62, 95% CI 1.13–2.31) [18].

The rate of placental abruption was significantly higher in the previous cesarean section group (1.3% vs. 0.3%, *p* = 0.0251), although absolute numbers remained low. This observation is consistent with established associations between prior cesarean delivery and increased placental abruption risk (22). However, literature comparing this outcome in the specific setting of IOL among women with versus without previous cesarean section is lacking.

Abnormal CTG was more frequent in women with a previous cesarean section (27.1% vs. 20.4%, *p* = 0.0058), accompanied by a higher rate of fetal blood sampling (8.7% vs. 5.0%, *p* = 0.0055). To date, no comparable studies have assessed these outcomes in a similar cohort. Similarly, arterial cord pH < 7.10 was more common in the previous cesarean section group (4.9% vs. 2.8%, *p* = 0.0381), although no differences were observed for pH < 7.00 (0% vs. 0.4%, *p* = 0.6171), base excess < −12 (2.3% vs. 2.4%, *p* = 0.8428), or base excess < −16 (0.3% vs. 0.3%, *p* = 1.0). Locatelli et al. also reported no significant differences in pH < 7.0 (0% vs. 0.5%, *p* = 0.46) (8), although other detailed neonatal outcomes were not analyzed in that study.

The Apgar score < 5 at 5 min did not differ between groups. This concurs with prior findings comparing women with previous cesarean section undergoing IOL and those without such history (0.3% vs. 0.5%, *p* = 0.93) [10]. Similarly, studies comparing IOL to spontaneous labor onset in this population also found no difference in Apgar < 5 or < 7 [14]. One prospective study reported significantly higher rates of Apgar < 7 in women with prior cesarean section and IOL (9.6% vs. 1.5%, *p* = 0.01) [23], though the sample size in that subgroup was small (n = 52), limiting generalizability.

Shoulder dystocia occurred more frequently in the previous cesarean section group (2.3% vs. 0.8%, *p* = 0.0265). However, absolute numbers were small (n = 7 vs. n = 26), and no existing studies have specifically addressed this outcome in comparable cohorts. Ashwal et al. did not observe significant differences in shoulder dystocia when comparing IOL and spontaneous labor among women with previous cesarean section (0.8% vs. 0.6%, *p* = 0.67) [14].

Transfer rates to neonatal care units were not significantly different (10% vs. 11.5%, *p* = 0.4328), which is in line with other retrospective analyses showing comparable rates between women with previous cesarean section undergoing IOL and those with spontaneous labor (6.2% vs. 5.9%, *p* = 0.77) [14].

The overall cesarean section rate was virtually identical in both groups (14.8% vs. 14.9%, *p* = 0.9692). This is notably lower than rates reported in other studies involving IOL in women with prior cesarean section, which range from 31.5 to 40.1% [12, 18, 21]. Locatelli et al. found higher cesarean section rates in women with previous cesarean (29.0% vs. 13.8%, *p* < 0.001) [10], and Ashwal et al. reported 16.2% in the IOL group compared to 10.7% with spontaneous labor (*p* = 0.01) [14]. These differences may reflect variation in induction protocols; notably, all IOL cases in our study were initiated with cervical ripening via CRB. Moreover, the comparatively low cesarean section rate observed in our cohort may be attributed to the strict selection criteria applied: the only obstetric risk factor present in the study population was a history of cesarean section, while other relevant risk factors such as hypertensive disorders, gestational diabetes, fetal growth abnormalities, abnormal fetal monitoring, or placenta previa were explicitly excluded. This results in a particularly low-risk population, which is not fully comparable to most previously published cohorts. The high rate of successful vaginal deliveries and low rates of severe neonatal outcomes in both groups likely reflect this highly selected setting.

The rate of vaginal operative delivery was significantly higher in the previous cesarean section group (17.8% vs. 11.9%, *p* = 0.0052). This is lower than previously reported rates for women with prior cesarean section and IOL (21.6–22.2%) [12, 21], but higher than the 5.6% found in a meta-analysis of 282 women [18]. Discrepancies likely stem from differences in study design and populations.

Although spontaneous vaginal delivery was less frequent in women with a prior cesarean section (82.2% vs. 88.1%, *p* = 0.0052), the success rate of 82.2% remains high. This exceeds the 37.8% rate reported in another retrospective analysis [12]. Most studies report lower vaginal delivery rates following IOL in this population (58.2–66.4%) [12, 18, 22, 23].

Importantly, the primary aim of this study was to assess the overall maternal and perinatal risk profile associated with induction of labor, rather than to evaluate rare single complications in isolation. The use of a composite outcome was therefore intended to capture clinically relevant events that are more frequent and thus more informative for daily clinical decision-making.

Some of the observed differences in secondary outcomes, such as placental abruption, abnormal cardiotocography, shoulder dystocia, and fetal blood sampling, are based on small absolute numbers of events. In the context of multiple comparisons, these findings may be subject to type I error and should therefore be interpreted with caution. They should be considered exploratory and hypothesis-generating, requiring confirmation in larger studies.

The applied exclusion criteria resulted in a selected low-risk population, which may limit generalizability. However, this approach was chosen deliberately to reduce confounding and to allow a more focused assessment of the effect of induction of labor itself. Consequently, our findings primarily apply to a selected low-risk population and should be interpreted with caution when extrapolating to routine clinical practice.

Limitations of this study include its retrospective nature. As a retrospective study, the analysis is subject to potential sources of bias, including selection bias and residual confounding, despite the applied inclusion and exclusion criteria. An additional limitation of our study is the use of different induction methods between groups. While this reflects routine clinical practice and guideline-based management—particularly the avoidance of misoprostol in women with a prior cesarean section—it introduces clinical heterogeneity. Therefore, the observed differences between groups may partly be influenced by differences in induction methods rather than uterine scar status alone.

Strengths include the standardized induction protocol over a ten-year period and comprehensive assessment of both maternal and neonatal outcomes. To our knowledge, only one comparable study [10] has analyzed both groups—women with and without prior cesarean section requiring IOL—though it focused on fewer outcome parameters.

Most existing literature compares IOL in women with prior cesarean section to elective repeat cesarean or spontaneous labor. Future prospective studies should directly compare maternal and neonatal outcomes in women with prior cesarean undergoing IOL versus those without prior cesarean in the same clinical setting to better guide clinical counseling and decision-making.

A key limitation of the present study is the insufficient statistical power to evaluate rare but clinically critical outcomes such as uterine rupture. Based on reported rupture rates of approximately 0.5–1%, a substantially larger cohort would be required to detect meaningful differences between groups. The absence of uterine rupture cases in our cohort should therefore not be interpreted as evidence of safety.

## Conclusion

In this retrospective cohort study, the overall risk of adverse maternal and perinatal outcomes did not differ significantly between women undergoing labor induction with or without a history of cesarean section. However, women with a prior cesarean section more frequently exhibited abnormal cardiotocography, required invasive fetal assessment, and experienced higher rates of operative vaginal delivery, placental abruption, and shoulder dystocia. These findings suggest that labor induction after a previous cesarean section, while not associated with an increased overall composite risk, should not be regarded as a low-risk scenario. Instead, it requires careful monitoring and should be conducted in settings equipped for immediate obstetric and neonatal intervention. Importantly, these findings are based on a selected low-risk cohort and should therefore be interpreted with caution when extrapolating to higher-risk obstetric populations.

## Data Availability

The datasets generated during the current study are available from the corresponding author on reasonable request.
